# Occurrence of ergot alkaloids and deoxynivalenol in Brazilian wheat and cattle feed determined by UHPLC-MS/MS

**DOI:** 10.1007/s12550-026-00675-9

**Published:** 2026-08-28

**Authors:** Cristiane Rosa da Silva, Cristina Tonial Simões, Luara Medianeira de Lima Schlösser, Isadora Fabris Laber, Luriane Medianeira Carossi Leal, Laura Bonai Casal, Carlos Alberto Araújo de Almeida, Carlos Augusto Mallmann

**Affiliations:** 1 Department of Preventive Veterinary Medicine, Laboratory of Mycotoxicological Analyses, Federal University of Santa Maria, Santa Maria, Rio Grande do Sul 97105-970 Brazil; 2 Department of Clinical and Toxicological Analyses, Federal University of Santa Maria, Santa Maria, Rio Grande do Sul 97105-970 Brazil

**Keywords:** Cattle feed, Ergot alkaloids, Food safety, Wheat

## Abstract

This study presents a novel and comprehensive assessment of the presence of ergot alkaloids (EAs, ergocristine, ergocornine, α-ergocryptine, ergotaminine, and ergosine) and deoxynivalenol (DON), individually and in combination, in wheat and cattle feed in Brazil. A total of 92 wheat grain and 79 cattle feed samples from different feed and food Brazilian industries were analyzed by ultra-high-performance liquid chromatography coupled with tandem mass spectrometry (UHPLC-MS/MS) between January 2023 and March 2025. Approximately 78% of wheat samples and 72% of cattle feed samples contained at least one EA. Ergosine was the predominant alkaloid in wheat, with a mean concentration of 8.06 µg/kg. In contrast, ergotaminine was the primary contaminant in cattle feed, with a mean concentration of 2.37 µg/kg. DON also showed high contamination levels, with mean concentrations of 1,913 µg/kg in wheat and 222.14 µg/kg in cattle feed. All five EAs were simultaneously detected in 8/92 wheat samples, whereas no simultaneous occurrence of all five EAs was observed in cattle feed samples. The co-occurrence of DON and different EAs ranged from 14.1 to 65.2% in wheat and from 6.3 to 40.5% in cattle feed. These findings highlight the importance of mycotoxin monitoring in Brazil and demonstrate the robustness of UHPLC-MS/MS as a tool for detecting these mycotoxins in complex matrices such as animal feed.

## Introduction

Fungal contamination of cereal crops is a major concern worldwide: an estimated 25–50% of cereals are contaminated, and 5–10% are deemed unsafe for consumption, leading to substantial economic losses (Alizadeh et al. 2022). Mycotoxin production is among the most critical consequences of this contamination, as these toxic compounds compromise food and feed safety, threatening public health. Therefore, continuous monitoring and robust analytical methods are necessary to accurately quantify contamination levels and assess associated risks in agricultural commodities (Pörtner et al. 2022).

Global wheat production is projected at 842.2 million metric tons in the 2025/2026 harvest, with total wheat utilization potentially reaching 805.1 million metric tons, driven by population growth and rising food demand (FAO, 2025). However, drastic climate changes (e.g., rising temperatures, more frequent extreme weather events, and altered precipitation patterns) are likely increasing pressure on wheat production and global food safety (Tkachenko et al. 2021). Recently, the frequency and intensity of these events have further increased the adverse effects on food safety (IPCC 2023).

Alkaloids are mycotoxins with alkaline properties, primarily produced by *Claviceps spp*., and commonly found in cereal crops such as barley, rye, and wheat (Poapolathep et al. 2021). In humans, acute toxicity of ergot alkaloids (EAs) can cause several adverse effects, including fever, gangrene, abortion, lactation suppression, and hypersensitivity reactions (Haque et al. 2020). Chronic ergotism is characterized by gangrenous necrosis of the extremities and gastrointestinal disturbances. In animals, severe EA-induced vasoconstriction can lead to ischemia and necrosis in the ears, tails, and hooves (Stoev 2024).

Deoxynivalenol (DON), a type B trichothecene primarily produced by *Fusarium graminearum*, is the most frequently detected mycotoxin in Brazilian wheat and wheat by-products, making it a major contaminant of concern in this commodity (Del Ponte et al. 2012; Mallmann et al. 2017). Consequently, DON is also frequently detected in livestock feed (Twarużek et al. 2021), particularly in cattle diets, where wheat and wheat by-products are commonly used. In both humans and animals, DON often causes gastrointestinal disturbances, such as nausea, vomiting, and diarrhea (Marc 2022). Therefore, the co-occurrence of EAs and DON warrants attention due to the distinct mechanisms of action of these mycotoxins. While DON is primarily associated with inhibition of protein synthesis, impaired intestinal integrity, and modulation of the immune response, EAs act predominantly through vasoconstrictive and neuroendocrine mechanisms, suggesting potential combined toxic effects that remain poorly understood (Stoev 2023). This complexity is illustrated by the European Food Safety Authority (EFSA Contam Panel et al., 2024), which reported that the interpretation of adverse effects in animals may be complicated by the simultaneous occurrence of multiple mycotoxins in feed, including a case of tail necrosis in rabbits associated with the concomitant presence of EAs, DON, T-2 toxin, and zearalenone.

Under European Regulation (EU) no. 2023/915 (EC, 2023a), the maximum tolerated level (MTL) for EAs in wheat intended for human consumption is 150 µg/kg, based on the lower bound sum of ergocornine/ergocorninine, ergocristine/ergocristinine, ergocryptine/ergocryptinine (α- and β-forms), ergometrine/ergometrinine, ergosine/ergosinine, and ergotamine/ergotaminine. Additionally, EU n°. 2024/1022 (EC, 2024) sets the MTL for DON in wheat intended for human consumption at 1,000 µg/kg. For animal feed, Commission Recommendation (EU) no. 2016/1319 (EC, 2016) provides guidance values of 8 mg/kg for cereals and cereal products intended for animal feed and 5 mg/kg for compound feed; no EU maximum limit for EAs in animal feed has yet been established. Additionally, in Brazil, the National Health Surveillance Agency (ANVISA, 2022) Normative Instruction n°. 160/2022 sets the MTL of 2,000 µg/kg for DON in wheat grain intended for human consumption, but no Brazilian regulatory limit currently exists for DON or EAs in animal feed, as well as for EAs in human food.

Although regulatory limits have traditionally been established considering individual mycotoxins, the natural contamination of cereals and feed frequently involves the simultaneous occurrence of multiple metabolites due to the colonization of the same crop by different fungal genera, such as *Claviceps* and *Fusarium* (Ahmed et al. 2022; Van den Brand et al. 2022). Consequently, humans and animals are usually exposed to multiple compounds, representing a growing concern for public and animal health (Stoev 2023). Considering that current risk assessment approaches remain predominantly based on individual mycotoxins, co-occurrence studies are essential to support cumulative risk assessment and the development of more comprehensive regulatory strategies for food and feed (Cattaneo et al. 2023).

In Brazil, data on the occurrence and co-occurrence of EAs, as well as their association with other mycotoxins, remain scarce, and no consolidated datasets are currently available for consistent national or international comparisons. Therefore, the present study aimed to assess the occurrence of five EAs and DON in Brazilian wheat and cattle feed using a validated ultra-high-performance liquid chromatography-tandem mass spectrometry (UHPLC-MS/MS) method, with emphasis on their natural co-occurrence patterns.

## Materials and methods

### Chemical reagents

The chemical reagents used in the present study were HPLC grade (> 98% purity). Acetonitrile (ACN), ammonium acetate, formic acid, and methanol were obtained from J.T. Baker (Phillipsburg, NJ, USA). Ultrapure water was produced using a Milli-Q A10 system (Millipore, Bedford, MA, USA). A solution of 1.0 M ammonium acetate was prepared by dissolving 7.708 g of ammonium acetate into 100 mL of ultrapure water. The analytical standards of ergocornine, α-ergocryptine, ergocristine, ergotaminine, and ergosine were purchased from Romer Labs (Tulln, Austria). The certified reference standards were selected based on their availability at the time of this study. Additional EAs reference standards, including ergotamine, could not be obtained because of regulatory restrictions affecting the importation of certified reference materials into Brazil. Primary and secondary amine (PSA) sorbents and C18 silica were obtained from Merck (Darmstadt, Germany). Filtration was carried out using a polytetrafluoroethylene (PTFE) syringe filter supplied by Whatman (Maidstone, UK). The certified standard of DON was obtained from Sigma-Aldrich (St. Louis, MO, USA).

### Wheat and feed samples

Between January 2023 and March 2025, a total of 92 wheat grain samples and 79 complete pelleted cattle feed samples (for both beef and dairy cattle) from Brazilian flour mills, feed manufacturers, and grain processing industries were sent for analysis at the Laboratory of Mycotoxicological Analysis, Federal University of Santa Maria, Brazil. The samples originated from companies located in the Brazilian states of Rio Grande do Sul, Santa Catarina, Paraná, and São Paulo, which are recognized as major contributors to both Brazilian wheat production and livestock farming, particularly dairy cattle production (CONAB, 2026; CILeite, 2026). The samples were submitted by companies as part of their routine mycotoxin monitoring programs, and, to ensure confidentiality, company names are not disclosed. The participating companies were requested to follow the sampling methods described by Whitaker et al. (2011). In the laboratory, samples (with at least 500 g) were ground at 1 mm using a ZM200 ultra-centrifugal mill (Retsch GmbH, Haan, Germany) and subsequently homogenized. Afterwards, samples were prepared for the quantification of ergosine, ergocornine, α-ergocryptine, ergotaminine, ergocristine, and DON.

### Sample preparation

### Deoxynivalenol

For the determination of DON, an adapted method based on Berthiller et al. (2005) was applied. A 3 g portion of the sample was placed in a Falcon tube and extracted with 24 mL of methanol–water (70:30, v/v), followed by vortex mixing for 20 min using an MA563 orbital shaker. The resulting extract was centrifuged at 1258 × g for 5 min at 20 °C. A 40 µL aliquot of the supernatant was diluted directly in a glass vial with 960 µL of a solution composed of 50% water-ammonium acetate (99:1, v/v) and 50% methanol-water-ammonium acetate (90:9:1, v/v/v) prior to chromatographic analysis.

### UHPLC-MS/MS conditions

#### Ergot alkaloids

The quantification of the five EAs was performed by UHPLC-MS/MS. The instrumental setup consisted of a 1290 Infinity UHPLC system (Agilent Technologies Inc., Santa Clara, CA, USA) connected to a 5500 QTRAP mass spectrometer (Sciex, Framingham, MA, USA) operating with electrospray ionization (ESI). Chromatographic separation was performed at 40 °C using a Gemini C18 column (150 × 2.0 mm, 5.0 μm particle diameter) (Phenomenex, Torrance, CA, USA).

The mobile phase was composed of solvent A (water containing formic acid and ammonium acetate at 998:1:1, v/v/v), and solvent B consisted of pure ACN. The gradient program was as follows: solvent B was increased linearly from 1% at 0 min to 100% at 12 min, maintained at 100% from 12.01 to 15 min, returned to 1% at 15.01 min, and held at 1% until 19 min for column re-equilibration. A sample volume of 10 µL was injected, with the flow rate set to 0.5 mL/min.

The mass spectrometer was operated in positive ionization mode under the following conditions: ion source temperature 700 °C; curtain gas (N₂) 20 psi; collision gas (N₂) 5 psi; ion spray voltage 5000 V; and nebulizer gas and drying gas supplied with nitrogen were set to 50 psi.

The validated method included the simultaneous determination of five EAs (ergosine, ergocornine, α-ergocryptine, ergocristine, and ergotaminine), selected according to the availability of certified analytical reference standards during method development. Data acquisition was performed in multiple reaction monitoring (MRM) mode using one transition for quantification and one transition for confirmation for each analyte. The monitored precursor/product ion transitions, retention times (RT), declustering potentials (DP), collision energies (CE), and collision cell exit potentials (CXP) were as follows: ergosine, *m/z* 548.3→223.2 (quantifier) and 548.3→208.2 (qualifier), RT 8.93 min, DP 56 V, CE 45/57 V, and CXP 14/12 V; ergocornine, *m/z* 562.2→223.2 and 562.2→208.2, RT 9.07 min, DP 56 V, CE 47/63 V, and CXP 12/12 V; α-ergocryptine, *m/z* 576.3→223.2 and 576.3→208.2, RT 9.21 min, DP 51 V, CE 47/61 V, and CXP 14/16 V; ergocristine, *m/z* 610.4→592.4 and 610.4→223.2, RT 9.27 min, DP 56 V, CE 21/47 V, and CXP 18/12 V; and ergotaminine, *m/z* 582.2→223.2 and 582.2→208.2, RT 9.03 min, DP 66 V, CE 47/59 V, and CXP 12/14 V. The simultaneous determination of parent EAs and their corresponding epimers was not included in the validated method. The analytical conditions described above correspond to the UHPLC-MS/MS method developed and validated by Silva et al. (2025).

#### Deoxynivalenol

Quantification of DON was performed using an Agilent 1290 Infinity UHPLC system coupled to a Sciex 5500 QTRAP tandem mass spectrometer equipped with ESI source. Chromatographic separation was achieved using a Zorbax SB-C18 column (4.6 × 150 mm, 5 μm particle diameter) (Agilent Technologies Inc., Santa Clara, CA, USA) at 40 °C. The mobile phases consisted of water–ammonium acetate (99:1, v/v) and methanol–water–ammonium acetate (90:9:1, v/v/v). The gradient program was applied as follows: 10% B from 0 to 2 min; 50% B from 2.01 to 7 min; 80% B from 7.01 to 8 min; 100% B from 8.01 to 13 min, followed by re-equilibration with 10% B from 13.01 to 17 min. The injection volume was 10 µL, with a constant flow rate of 1 mL/min.

The mass spectrometer operated in negative ESI mode under the following conditions: ion source temperature 700 °C; curtain gas (N₂) 20 psi; collision gas (N₂) 9 psi; ion spray voltage − 4500 V; nebulizer gas and drying gas supplied with nitrogen were set at 45 psi. Data acquisition was performed in MRM mode using the transitions *m/z* 295→265 for quantification and *m/z* 295→138 for confirmation, with a RT of 3.5 min. The DP was − 65 V for both transitions, whereas the CE was 57 V and 43 V, and the CXP was 5 V and 9 V for the quantifier and qualifier transitions, respectively. These analytical conditions correspond to the method described by Mallmann et al. (2017).

### Method performance parameters

The analytical methods were previously validated according to EURACHEM (2014) and European Commission guidelines (EC, 2023b). Validation parameters included limit of detection (LOD), limit of quantification (LOQ), linearity, matrix effect, precision, recovery, reproducibility, selectivity, and uncertainty.

LOD and LOQ were established based on signal-to-noise ratios of 3:1 and 10:1, respectively, using spiked standards. For DON, LOD and LOQ were 100 and 200 µg/kg, respectively. For the five EAs (ergosine, ergocornine, α-ergocryptine, ergocristine, and ergotaminine), LOD and LOQ were 0.5 and 1 µg/kg, respectively.

Linearity was verified through external calibration curves for all analytes. For DON, a seven-point curve ranging from 200 to 8,000 µg/kg was used, based on a method adapted from Berthiller et al. (2005), yielding a correlation coefficient (R²) of 0.9998. For the EAs, a six-point curve ranging from 0.5 to 500 µg/kg was used, as previously validated by Silva et al. (2025), with correlation coefficients (r) of 0.9993 for ergosine, 0.9996 for ergocornine, 0.9995 for α-ergocryptine, 0.9996 for ergocristine, and 0.9998 for ergotaminine.

Matrix effects were evaluated for all analytes by comparing calibration curves prepared in solvent and in matrix, using the F-test based on analysis of variance homogeneity (MAPA, 2015). For DON, the calculated F value (3.40) was lower than the tabulated F value (11.39), indicating no significant matrix effect within the tested range. For the EAs, the calculated F values were 2.87, 2.06, 2.75, 3.01, and 1.02 for ergosine, ergocornine, α-ergocryptine, ergocristine, and ergotaminine, respectively. All calculated F-values were lower than the tabulated F value (10.67), confirming no significant matrix effect.

Recovery was assessed at three fortification levels, with results within the acceptance range of 70–120%. Precision (repeatability and intermediate precision) showed relative standard deviations (RSDs) below 20%, in line with recommendations. Selectivity was confirmed by the absence of interfering peaks in blank chromatograms. Expanded measurement uncertainty was estimated considering both precision and bias and applying a coverage factor of k = 2 (95% confidence level).

### Statistical analyses

Data were analyzed using descriptive statistics in Statgraphics Centurion XV (Statgraphics Technologies, Inc., The Plains, VA, USA). The following descriptive measures were calculated for each mycotoxin: mean, standard deviation, minimum, and maximum values.

### Ergot alkaloids

The methodology validated by Silva et al. (2025) was followed. From the homogenized samples, 1 g was transferred to Falcon tubes and extracted with 10 mL of solvent composed of 75% ACN and 25% water, containing 0.1% formic acid. The suspension was shaken for 5 min in an MA563 orbital shaker (Marconi, São Paulo, Brazil). Subsequently, samples were centrifuged (Eppendorf, Hamburg, Germany) at 1258 × g for 5 min at 18 °C. The supernatant fraction was transferred to tubes containing 50 mg of PSA and 50 mg of C18 sorbent, vortex-mixed for 1 min, and centrifuged under the same previous conditions. The cleaned extract was dried under a stream of nitrogen at 40 °C. The dried residue was subsequently resuspended in 1,000 µL of a 1:1 (v/v) mixture of aqueous and organic solvents. The aqueous component was composed of water, formic acid, and ammonium acetate (998:1:1, v/v/v), while the organic component consisted of pure ACN. The final solution was passed through a 0.22 μm nylon syringe filter and transferred to an amber vial for chromatographic analysis.

## Results and discussion

Although several studies have described HPLC-based methods for the determination of EAs, as well as their occurrence in a variety of food and feed matrices worldwide, only a limited number of studies have reported the implementation of this analytical methodology for EAs quantification in Brazil (Abreu et al. 2023a, b; Silva et al. 2025). One of the studies focused on cocoa beans, and no EAs were detected in the analyzed samples (Abreu et al. 2023b). Consequently, despite a few reports of ergotism in cattle following the ingestion of feed contaminated with *C. purpurea* (Ilha et al. 2003; Hemckmeier et al. 2018), there is still a lack of data on the occurrence of EAs in Brazilian wheat and animal feed, and the contamination profile of these mycotoxins remains unknown.

A total of 92 wheat grain samples were analyzed for the presence of EAs and DON between 2023 and 2024; results are presented in Table 1. Occurrence, concentration ranges, general mean, standard deviation, and mean of the positive samples are reported for each mycotoxin. Approximately 78% (*n* = 72) of the wheat samples contained at least one detectable EA. Previous studies support this prevalence. In Italy, 62 of 71 (87%) wheat samples were contaminated with at least one of 12 EAs (Debegnach et al. 2019). Similarly, Shi and Yu (2022) reported that 84% of wheat samples processed for industrial use tested positive for at least one EA, while a survey across European countries found EAs in 85% of samples (Arroyo-Manzanares et al. 2018). An even higher occurrence was observed in western Canada, where all wheat samples contained at least one EA (Tittlemier et al. 2015).

**Table 1:** Occurrence of deoxynivalenol (DON), ergocornine, ergocristine, α-ergocryptine, ergosine, and ergotaminine in wheat samples from 2023 to 2024

Mycotoxins	Samples (*n*)	Occurrence (%)	Range (µg/kg)	Mean (µg/kg)	SD^1^	Mean of positives (µg/kg)
DON	92	97.8	< LOQ^2^ – 3,490	1,913	791.7	1,994
Ergosine	92	68.5	< LOQ – 129.0	8.06	17.5	11.7
Ergotaminine	92	45.6	< LOQ – 1,700	45.6	225.8	99.9
α-Ergocryptine	92	38.0	< LOQ – 63.9	2.97	9.00	7.79
Ergocornine	92	27.1	< LOQ – 58.0	2.42	8.25	8.89
Ergocristine	92	13.0	< LOQ – 99.8	1.94	10.9	14.8

^1^SD = Standard deviation calculated using all analyzed samples

^2^LOQ = Limit of quantification

In this study, ergosine was the most frequently detected alkaloid (68.5%), followed by ergotaminine (45.6%), α-ergocryptine (38.0%), ergocornine (27.1%), and ergocristine (13.0%). The high prevalence of ergosine aligns with findings of studies conducted in Europe by Malysheva et al. (2014) and Rollo et al. (2025). In contrast, Shi and Yu (2022) observed higher occurrences for ergocristine (66%) and ergocryptine (61%) in wheat from Canada, which may reflect differences in agricultural practices, soil conditions, environmental temperature, and precipitation, factors known to influence EAs incidence (Tittlemier et al. 2015).

Mean concentrations of EAs were 8.06, 45.6, 2.97, 2.42, and 1.94 µg/kg for ergosine, ergotaminine, α-ergocryptine, ergocornine, and ergocristine, respectively. Concentration levels reached up to 1,700 µg/kg, with the highest value attributed to ergotaminine, indicating its predominance in the contamination profile. Similar concentrations were reported by Shi and Yu (2022), whereas Carbonell-Rozas et al. (2023) found total EAs concentrations in wheat ranging from 3.66 to 76.0 µg/kg.

The high occurrence of DON (97.8%) observed in the present study confirms the status of this trichothecene as one of the main wheat contaminants (Del Ponte et al. 2012; Mallmann et al. 2017), with concentrations up to 3,490 µg/kg and a mean of 1,994 µg/kg in the positive samples. These values were higher than those reported by Machado et al. (2017), who detected DON in 66.5% of samples in Brazil, with a mean concentration of 1,047 µg/kg. Correspondingly, Santos et al. (2011) reported a 72.2% occurrence of DON in Brazilian wheat, with levels up to 1,592 µg/kg. DON was also highly prevalent in other countries; studies in China found detection rates of 90.8% (Zhao et al. 2021) and 94.4% (Luan et al. 2025). These findings underscore the toxicological relevance of DON in Brazilian wheat, corroborating previous evidence identifying it as one of the most prevalent mycotoxins worldwide.

The co-occurrence of mycotoxins may increase toxicological effects and should be considered in monitoring strategies (Luan et al. 2025). In the present study, frequent co-occurrence was observed between DON and EAs, as well as among different EAs (Table 2). Therefore, investigating potential synergistic effects is fundamental, as previous studies have shown correlations among different classes of mycotoxins in cereals (Blandino et al. 2017; Walkowiak et al. 2022). The highest co-occurrence was observed between DON and ergosine (65.2%), with concentrations of 2,073 µg/kg for DON and 12.1 µg/kg for ergosine. DON also co-occurred with the other four EAs, with results ranging from 46.7% for DON+ergotaminine to 14.1% for DON+ergocristine. Kim et al. (2024) reported similar co-occurrence between DON and EAs (73%) in cereal samples, reinforcing the importance of investigating the potential additive or synergistic effects of these mycotoxins. A higher co-occurrence in Chinese wheat was reported in a study by Luan et al. (2025), where 95.7% of the samples were contaminated by multiple mycotoxins, including DON and EAs.

**Table 2:** Co-occurrence of deoxynivalenol (DON), ergocornine, ergocristine, α-ergocryptine, ergosine, and ergotaminine in wheat samples from 2023 to 2024

Mycotoxins	Samples (*n*)	Co-occurrence (%)	Mean of first mycotoxin(µg/kg)	Mean of second mycotoxin (µg/kg)
DON + Ergosine	92	65.2	2,073	12.1
DON+ Ergotaminine	92	46.7	2,020	99.9
Ergosine + Ergotaminine	92	40.2	7.54	10.7
DON + α-Ergocryptine	92	39.1	2,074	1.79
DON + Ergocornine	92	28.2	2,057	8.88
Ergosine + α-Ergocryptine	92	32.6	19.4	8.19
Ergotaminine + α-Ergocryptine	92	27.2	164.3	10.0
α-Ergocryptine + Ergocornine	92	26.1	10.7	8.89
DON + Ergocristine	92	14.1	2,006	14.9
Ergocristine + Ergotaminine	92	13.0	7.17	13.5

The five EAs were simultaneously present in only 8 out of the 92 samples; however, relevant co-occurrences between two EAs were observed. Consistent with previous reports (Shi and Yu 2022), most wheat samples with detectable levels of ergosine also exhibited the presence of other EAs. The combination of ergosine and ergotaminine was observed in 40.2% of samples, with mean concentrations of 7.54 and 10.7 µg/kg, respectively. Ergosine also exhibited a relevant co-occurrence with α-ergocryptine, with the two mycotoxins being detected simultaneously in 32.6% of the samples. The ergotaminine + α-ergocryptine combination occurred in 27.2% of the samples, whereas α-ergocryptine+ergocornine and ergocristine+ergotaminine combinations occurred in 26.1% and 13.0% of the samples, respectively. The occurrence of multiple EAs in the present study was lower than that reported by Babič et al. (2020), who evaluated wheat samples from Slovenia and observed that approximately 25% of samples contained more than eight EAs, while 27% contained three to five EAs. However, direct comparisons among studies should be interpreted with caution, as the number of analytes investigated is different. Babič et al. (2020) analyzed 12 EAs, whereas the present study evaluated five EAs due to limitations in the availability and acquisition of analytical standards in Brazil. Consequently, studies investigating a greater number of analytes may report a higher EA co-occurrence rate. Nevertheless, despite this limitation, the present study provides relevant information on EAs occurrence in Brazil, where data regarding the contamination of food and feed ingredients with these mycotoxins remain scarce.

Wheat and wheat by-products are widely used as ingredients in cattle feed formulations. Therefore, commercial complete pelleted feed for dairy and beef cattle was also evaluated in the present study. However, human and livestock exposure to mycotoxins differs substantially due to distinct dietary patterns. Human diets are generally diverse, with wheat-derived products representing only one of many dietary components, thereby reducing the contribution of a single contaminated source to overall exposure. In contrast, cattle receiving compound feed, particularly during the finishing phase and semi-intensive production systems, may consume relatively uniform diets over extended periods, increasing the potential for chronic exposure. In Brazil, cattle production remains predominantly pasture-based, whereas concentrate-based diets are more commonly used during the finishing phase and in semi-intensive systems (Nunes et al. 2024). Therefore, despite representing only a portion of the national herd, these systems represent relevant exposure scenarios for mycotoxin-contaminated feed.

Several investigations have documented the occurrence of EAs in cereals and animal feed across different countries, demonstrating that contamination by these compounds is geographically widespread. In developing countries, exposure to EAs remains a significant challenge, associated with negative impacts on livestock health and performance (Schummer et al. 2020). However, data on EAs animal feed in Brazil are still scarce. Table 3 presents the occurrence and levels of DON, ergocornine, ergocristine, α-ergocryptine, ergosine, and ergotaminine in cattle feed samples analyzed between 2024 and 2025.

**Table 3:** Occurrence of deoxynivalenol (DON), ergocornine, ergocristine, α-ergocryptine, ergosine, and ergotaminine in cattle feed samples from 2024 to 2025

Mycotoxins	Samples (*n*)	Occurrence (%)	Range (µg/kg)	Mean (µg/kg)	SD^1^	Mean of positives (µg/kg)
DON	79	65.8	< LOQ^2^ − 1,040	222.1	159.9	337.6
Ergotaminine	79	59.5	< LOQ – 15.4	2.37	3.47	3.98
Ergosine	79	39.2	< LOQ – 10.1	1.15	2.00	2.94
α-Ergocryptine	79	7.6	< LOQ – 2.69	0.13	0.48	1.72
Ergocristine	79	1.2	< LOQ – 3.25	0.04	0.36	3.25
Ergocornine	79	0.0	-	-	-	-

^1^SD = standard deviation calculated using all analyzed samples

^2^LOQ = Limit of quantification

More than 72% (*n* = 57) of the cattle feed samples contained at least one detectable EA. Among these compounds, ergotaminine was the most prevalent, with an occurrence of 59.5% and concentrations from < LOQ to 15.40 µg/kg. Nevertheless, the observed concentrations were lower than those reported in studies conducted in Europe. Ruhland and Tischler (2008) reported the presence of EAs in 86–100% of feed samples collected in Germany, with mean contamination levels ranging from 25 to 96 µg/kg. Beyond the differences in the number of analytes evaluated across studies, as previously discussed, the different results among studies may also be associated with variations in the analytical methods employed. While the German group used fluorescence detection, the UHPLC-MS/MS was applied herein, a technique recognized for its higher selectivity and sensitivity. This methodology reduces matrix interference and improves accuracy in detecting compounds at low concentration levels.

Similarly, a survey conducted in Netherlands reported an EA occurrence of 83% in animal feed samples, with an average concentration of 89 µg/kg and a maximum concentration of 1,231 µg/kg, using LC–MS/MS (Mulder et al. 2012). Furthermore, according to Malysheva et al. (2014), EAs occurrence in feed was strongly influenced by the type of cereal used in food and feed formulations. In that study, EAs were present in 84% of rye-based food, 67% of wheat-based food, 52% of rye-based feeds, 48% of multigrain food, and 27% of wheat-based feeds, with total concentrations ranging from ≤ 1 to 12,340 µg/kg. These results indicate that feed formulations with higher proportions of rye and wheat tend to present higher contamination levels. Ergosine was the second most prevalent EA in cattle feed, being detected in 39.2% of the samples, with mean levels of 1.15 µg/kg and concentrations reaching up to 10.1 µg/kg. Other EAs presented lower occurrence. α-ergocryptine was detected in only 7.6% of the samples, with low mean levels (0.13 µg/kg), whereas ergocristine occurred in only 1.2% of the samples and ergocornine was not detected. In the study by Malysheva et al. (2014), ergosine was the most prevalent EA, quantified in 19% of samples. In the final dataset, composed of feed samples collected between 2011 and 2016 across five European countries, 50% of samples did not contain detectable levels of EAs above 0.1 µg/kg.

The toxicity of EAs is not uniform across the compounds monitored in this study and should be considered when interpreting the results. (R)-epimer forms are considered more toxicologically active than their corresponding (S)-epimer forms, since epimerization is associated with reduced receptor binding affinity. However, among the (S)-epimers specifically, ergotaminine has demonstrated the greatest vasoconstrictive potency in vitro, producing a stronger contractile response than ergocorninine, ergocristinine, or ergocryptinine (Cherewyk et al., 2020). This finding is particularly relevant to the present results, as ergotaminine was the predominant EA detected in cattle feed (59.5%) and the second most prevalent EA in wheat (45.6%). Furthermore, since (R)- and (S)-epimers can interconvert depending on environmental conditions such as temperature and pH, the presence of ergotaminine should not be interpreted as representing a negligible toxicological relevance.

DON was detected in 65.8% of the cattle feed samples, with a mean concentration of 222.1 µg/kg and levels ranging from < LOQ to 1,040 µg/kg. In Brazil, a five-year survey reported that 97% of bovine feeds were contaminated with at least one mycotoxin, and DON was the most frequently detected mycotoxin, occurring in 67.8% of samples (Biscoto et al. 2022). Additionally, in a study conducted in Poland between 2021 and 2024, which analyzed 1,102 feed samples—including formulations intended for cattle—DON showed a 100% occurrence, with concentrations reaching up to 11,033 µg/kg (Skrzydlewski et al. 2025).

The co-occurrence of DON and EAs in cattle feeds is presented in Table 4. DON co-occurred with ergotaminine, ergosine, and α-ergocryptine in 40.5, 31.6, and 6.3% of the cattle feed samples, respectively. Contamination levels of DON when co-occurring with EAs ranged from 351.5 to 515.4 µg/kg. Among EAs, the combination of ergosine and ergotaminine was the most common, being detected in 26.6% of the samples, with mean concentrations in positive samples of 2.37 µg/kg and 5.09 µg/kg, respectively. The co-occurrence of α-ergocryptine with ergosine and ergotaminine was observed at lower frequencies (7.6% and 6.3%, respectively).

**Table 4:** Co-occurrence of deoxynivalenol (DON), ergocornine, ergocristine, α-ergocryptine, ergosine, and ergotaminine in cattle feed samples from 2024 to 2025

Mycotoxins	Samples (*n*)	Co-occurrence (%)	Mean of first mycotoxin (µg/kg)	Mean of second mycotoxin (µg/kg)
DON + Ergotaminine	79	40.5	364.1	4.42
DON + Ergosine	79	31.6	351.5	3.06
Ergosine + Ergotaminine	79	26.6	2.37	5.09
α-Ergocryptine + Ergosine	79	7.6	1.72	6.36
DON + α-Ergocryptine	76	6.3	515.4	1.73
α-Ergocryptine + Ergotaminine	79	6.3	1.79	7.95

A study conducted on rye-based feed reported similar co-occurrence proportions, including samples containing only a single pair of alkaloids (20%) and the simultaneous occurrence of all six evaluated toxins (23%). When analyzing the presence of specific EAs, the sum of ergometrine and ergometrinine was predominant, being detected in 38% of samples (Malysheva et al. 2014). Furthermore, a study conducted in Spain reported that among contaminated feed samples, 65% contained more than one EA; however, specific co-occurrence patterns were not detailed (Gámiz-Gracia et al. 2021). In this study, the highest contamination level was 27.9 µg/kg in a sample with 15.2 µg/kg of ergotaminine, 10.1 µg/kg of ergosine, and 2.69 µg/kg of α-ergocryptine.

Table 5 demonstrates the percentage of samples above the action or guidance levels for DON and EAs in wheat and/or cattle feed. Total EAs concentrations exceeding the EU regulatory MTL (150 µg/kg) for wheat were detected in only five (5.43%) of the 92 samples, suggesting isolated cases of harmful contamination. However, it should be noted that the total EA values reported in the present study are based only on the five quantified alkaloids and are not directly equivalent to the regulatory sum, which includes additional EAs and their corresponding epimers. Furthermore, 54.3% and 84.9% of the wheat samples contained DON concentrations above the Brazilian (2,000 µg/kg) and the EU action levels (1,000 µg/kg), respectively, indicating that contamination was not only frequent but also toxicologically relevant from a food safety perspective. None of the samples presented DON levels exceeding the guidance values (EC, 2016) established for cereals and cereal products intended for animal feed, as well as for compound feed.

**Table 5:** Action levels for deoxynivalenol and ergot alkaloids

Product	Mycotoxin	Description	Action/guidance levels, µg/kg	Samples above the action level, %	Analyzed levels, µg/kg
Wheat	Deoxynivalenol	Unprocessed cereal grains intended for human consumption^1^	1,000	84.8%	1,140–3,490
		Wheat grains intended for human consumption^2^	2,000	54.3%	2,020–3,490
		Cereals and cereal products intended for animal feed^3^	8,000	0%	-
	Ergot Alkaloids	Wheat intended for human consumption^4^	150	5.43%	201.4–2,050
Feed	Deoxynivalenol	Compound feed^3^	5,000	0%	-

^1^Commission regulation (EU) 2024/1022 amending regulation (EU) 2023/915 as regards maximum levels of deoxynivalenol in food. Off J Eur Union

^2^Normative Instruction No. 160, July 1, 2022 (Instrução Normativa No. 160, de 1 de julho de 2022). Establishes the maximum tolerated limits (MTLs) of contaminants in food. *Diário Oficial da União*, Brasília, Brazil

^3^Commission recommendation (EU) 2016/1319 amending recommendation 2006/576/EC as regards deoxynivalenol, zearalenone and ochratoxin A in pet food

^4^Commission regulation (EU) 2023/915 on maximum levels for certain contaminants in food and repealing regulation (EC) No 1881/2006. Off J Eur Union 119:103–157

In conclusion, by applying a previously validated UHPLC-MS/MS method, this study provided the first comprehensive assessment of EAs and DON simultaneous occurrence in Brazilian wheat and cattle feed, addressing a significant knowledge gap resulting from the limited availability of EAs monitoring capacity in the country. Nearly 78% of wheat samples and 72% of cattle feed samples contained at least one EA, with ergosine prevailing in wheat and ergotaminine in cattle feed. Co-occurrence of DON and EAs ranged from 14.1 to 65.2% in wheat and from 6.3 to 40.5% in cattle feed. These findings support the need for continuous monitoring and comprehensive risk assessment of mycotoxin contamination in Brazil. The results also confirm the robustness of UHPLC-MS/MS as a monitoring tool for these mycotoxins in complex matrices, such as animal feed.

## Data Availability

No datasets were generated or analysed during the current study.
